# Fetal DNA Causes Sex-Specific Inflammation From Human Fetal Membranes

**DOI:** 10.3389/fphys.2022.901726

**Published:** 2022-06-22

**Authors:** Chelsea A. Saito Reis, Po’okela K. Ng, Courtney Kehaulani Kurashima, Justin Padron, Claire Enid Kendal-Wright

**Affiliations:** ^1^ Natural Science and Mathematics, Chaminade University of Honolulu, Honolulu, HI, United States; ^2^ Department of Obstetrics, Gynecology and Women’s Health, John A. Burns School of Medicine, University of Hawaii, Honolulu, HI, United States

**Keywords:** fetal membranes, fetal DNA, inflammation, fetal sex, cytokine, methylation

## Abstract

Inflammation is central to the mechanisms of parturition, but the lack of understanding of how it is controlled in normal parturition hampers our ability to understand how it may diverge resulting in preterm birth. Cell-free fetal DNA is found in the amniotic fluid, and it is thought to be able to activate inflammation as a danger-associated molecular pattern. Although its levels increases with gestational age, its effect has not been studied on the human fetal membranes. Thus, the aim of this study was to determine if the fetal DNA can trigger inflammation in the human fetal membranes and, thus, potentially contribute to the inflammatory load. Isolated human amniotic epithelial cells and fetal membrane explants were treated apically with fetal DNA causing the translocation of NF-KB into the nucleus of cells and throughout the cells of the explant layers with time. Fetal membrane explants were treated apically with either small or larger fragments of fetal DNA. IL-6, TNFα, and GM-CSF secretion was measured by ELISA, and pro-MMP2 and pro-MMP9 activity was measured by zymography from apical and basal media. Increased apical IL-6 secretion and basal pro-MMP2 activity was seen with small fragments of fetal DNA. When the data were disaggregated based on fetal sex, males had significant increases in IL-6 secretion and basal increased activity in pro-MMP2 and 9, whereas females had significantly increased basal secretion of TNFα. This was caused by the smaller fragments of fetal DNA, whereas the larger fragments did not cause any significant increases. Male fetal DNA had significantly lower percentages of methylation than females. Thus, when the cytokine and pro-MMP activity data were correlated with methylation percentage, IL-6 secretion significantly correlated negatively, whereas GM-CSF secretion positively correlated. These data support the role of fetal DNA as an inflammatory stimulus in the FM, as measured by increased NF-κB translocation, cytokine secretion, and increased pro-MMP activity. However, the data also suggested that the responses are different from FM tissues of male and female fetuses, and both the fragment size and methylation status of the fetal DNA can influence the magnitude and type of molecule secreted.

## Introduction

Normal pregnancy concludes *via* the integration of several complex parturition pathways that coalesce in the human myometrium, cervix, and fetal membranes (FMs). However, these pathways and the specifics of their interactions are not fully understood. This knowledge gap not only hampers our ability to understand normal parturition mechanisms but also presents a significant barrier to our recognition of how they diverge into those pregnancies that terminate in preterm birth. What is currently clear is that regardless of the gestational age at the onset of parturition, inflammation is critical for its initiation (Rice, 2001). In normal pregnancies, this inflammation leads to changes in several maternal and fetal tissues but specifically results in FM weakening through the proinflammatory mediators that promote the production of cytokines (Kumar et al., 2006), apoptosis (Kumar et al., 2016), and increased matrix metalloproteinase (MMP) activity (Kumar et al., 2018). Indeed, the treatment of human FM, with interleukin-6 (IL-6), tumor necrosis factor alpha (TNFα), and granulocyte-macrophage colony-stimulating factor (GM-CSF), has been shown to directly weaken human FM (Kobayashi et al., 2010; Kumar et al., 2016).

The primary initiator of inflammation has not been established, but stretch (Kendal-Wright, 2007) and other amassing cell stressors have been implicated (Menon et al., 2016a; Sheller et al., 2016). Growing evidence also suggests that endogenous inflammatory mediators such as danger-associated molecular patterns (DAMPs) may contribute to this inflammation and its consequences (Menon et al., 2016b; Padron et al., 2020). Oxidative stress and hypoxia in the placenta cause the production of several DAMPs, including; uric acid, high mobility group box1 (HMGB1), S100 calcium-binding protein A (S100A), S100 calcium binding protein alpha-12 (S100A12), heat shock protein (HSP) 70 kD, and cell-free fetal DNA (cffDNA) (Baker et al., 2021).

Although little is known about the effects of cffDNA on the human FM, fetal DNA is readily found in the amniotic fluid (AF) where it is at much higher concentrations than in maternal plasma (Bianchi et al., 2001). The results from several studies show that there is no correlation between the amount of DNA in the AF and the maternal plasma, indicating that this source of fetal DNA is physiologically independent of the cffDNA circulating in the maternal vascular system (Hui and Bianchi, 2011). This is because the primary source of the nucleic acids in the AF is from the fetus (Larrabee et al., 2005) rather than from the placental trophoblast. However, they are both of fetal origin. Interestingly, the fetal DNA levels are high in the AF of premature preterm rupture of the membrane pregnancies with those who have a microbial-associated intrauterine infection (IAI) having the highest levels (Kacerovsky et al., 2018). On average, the size of the AF DNA is <200 bp, and it is thought to be more fragmented than that found in the maternal circulation (Burnham et al., 2020) but the methylation status can vary. This form of AF DNA is understood because its transcriptome characterization can be used to detect fetal genetic abnormalities. It is a useful, but an invasive diagnostic tool. Not only is it affected in normal pregnancies by gestational age, fetal maturity, and fetal sex, but also by pathologic states such as maternal obesity and various genetic syndromes (Park et al., 2021). The pregnancy complications of preeclampsia (Jung et al., 2019), intrauterine growth restriction (Cho et al., 2018) and preterm birth (Bhatti et al., 2021) have also been shown to be detectable through the AF transcriptome.

cffDNA released from the apoptotic and necrotic cells of placental origin into the maternal circulation has been studied more extensively (Kazemi et al., 2021). These DNA fragments are <313 bp (Sin et al., 2020) in length and present within the maternal circulation as early as 35 days gestation. They have been shown to increase throughout the pregnancy, peaking at term and then dropping after birth (Liu et al., 2007). Indeed, this source of cffDNA is also used for prenatal diagnostic detection of fetal chromosomal abnormalities (Wang et al., 2021) as it is less invasive than sampling from the AF. Increased circulating levels of cffDNA have also been associated with adverse pregnancy outcomes and pathologies such as; preeclampsia (Martin et al., 2014; Kwak et al., 2020), gestational diabetes (Hopkins et al., 2020), and preterm birth (Gomez-Lopez et al., 2020). cffDNA from the trophoblast is also known to increase by sterile inflammation, *via* HMGB1 (Yaganeh Kazemi et al., 2021), but its ability to generate inflammation itself is contentious. Some studies have clearly shown that it is able to interact with toll-like receptor 9 (TLR9) (Goldfarb et al., 2018) and that immune cells respond to it by producing inflammatory cytokines (Yeganeh Kazemi et al., 2021). However, others have not been able to confirm this (van Boeckel et al., 2020).

Like placental cffDNA, it is thought that fetal DNA in the AF could activate TLR9. Although much of the evidence for this comes from the work showing that mouse FM release cffDNA that is hypomethylated, can increase IL-6 through TLR9 *in vitro* (Sawyer et al., 2018). Interestingly, TLR9 is highly expressed in the cells of the human amnion (Sato et al., 2016). Thus, it is possible that in humans hypomethylated regions of fetal DNA bind to TLR9 expressed in the amnion to elicit an inflammatory signal that may contribute to the initiation of parturition. Therefore, the aim of this study was to determine if fetal DNA, similar to that which would be obtained from AF, can trigger inflammation in the human FM and thus potentially contribute to the inflammatory load central to the normal mechanisms of parturition.

## Materials and Methods

### Tissue Collection and Amnion Cell Culture

Term (≥38 week’s gestation) fetal membranes were collected from singleton, repeat Cesarean sections at Kapi’olani Medical Center for Women and Children (Honolulu, HI, United States) with approval from the Institutional Review Board (Hawaii Pacific Health). The reflected fetal membranes were isolated 1 inch from the placenta and washed in sterile phosphate-buffered saline (PBS) within 30 min of collection to remove blood. The primary amnion epithelial cells (AEC) were isolated as previously described (Kendal-Wright et al., 2010). Briefly, the primary amnion was stripped from the choriodecidua fetal membrane layers, and the amnion epithelial cells were subjected to four consecutive trypsin (0.2%) digestions (Gibco, Waltham, MA, United States) for 30 min at 37°C at 150 rpm. AECs were cultured in DMEM/F12 media (Gibco, Waltham, MA, United States) containing 10% fetal bovine serum (FBS, Gibco, Waltham, MA, United States), penicillin (100 U/mL), streptomycin (100 μg/ml) and incubated at 37°C in 95% air/5% CO_2_. AECs were utilized without passage upon reaching 90% confluence.

### Primary Explant System and Fetal DNA Treatment

The full thickness amnion choriodecidua fetal membrane integrity was assessed to ensure all layers were intact with no separation from other layers. The membranes were rinsed in sterile PBS and then cut into 2.5 cm^2^ × 2.5 cm^2^ pieces and placed onto sterilized Transwell frames (Corning Inc., Corning, NY, United States) without synthetic membrane. The two-compartment system was created by placing an elastic latex dental band around the tissue (Astern et al., 2013). The amnion side of the fetal membrane tissue faced apical to the Transwell insert creating the inner, upper well, while the decidua layer faced outward creating a completely separate, outer, lower well. Each mounted fetal membrane was placed in a single well within a 12-well tissue culture plate with DMEM/F12 medium on both sides of the membranes to allow equilibration for 24 h. The culture medium was removed and the apical side (upper well) of the fetal membrane compartment was treated with 100 ng/ml cffDNA in 500 µl DMEM/F12 media and the basal side (lower well) was supplemented with only 1.5 ml DMEM/F12 media for 24 h 1000 ng/ml of lipopolysaccharide (LPS, Sigma Aldrich, St. Louis, MO, United States) was also used to treat the apical well of the fetal membrane compartment system. The condition medium from the top and bottom wells were collected and centrifuged for 10 mins, 10,000 x g at 4°C to remove cell debris. The fetal membrane explant was removed from the Transwell apparatus and placed in 10% formalin (Epredia, Kalamazoo, MI, United States) for 24 h and then stored in sterile PBS until further analysis.

### DNA Isolation From Amnion Epithelial Cells, Sonication, and Verification

DNA isolation was performed using the QIAamp DNA Mini isolation kit (Qiagen, Hilden, Germany) from isolated primary AECs following the manufacturer’s instructions. DNA was quantified using a NanoDrop (Thermo Fisher Scientific, Waltham, MA, United States). To create smaller fragments of cffDNA that would be more similar to those seen in AF, it was sonicated after dilution to 10 ng/μl in AE buffer (Qiagen, Hilden, Germany) for 4 min within an ultra sonicating water bath (PS-20A, Vevor, Shanghai, China). cffDNA fragment size of the whole (non-sonicated) and sonicated samples were verified on a 2% agarose gel with ethidium bromide (Invitrogen, Waltham, MA, United States) and visualized under UV transillumination using a Chemidoc (Bio-Rad, Hercules, CA, United States).

### Immunocytochemistry

Primary isolated AECs (100,000 cells/well) were seeded into 4-well chamber slides and grown to 80% confluency. The AECs were treated with 0, 1, 10, 100, and 1,000 ng/ml 4 min sonicated cffDNA or 1,000 ng/ml of lipopolysaccharide (LPS). After treatment, the AECs were fixed with 4% paraformaldehyde (PFA) (Sigma Aldrich, St. Louis, MO, United States) in 1X PBS for 15 mins, followed by two washes of 1X PBS. Non-specific binding was blocked with 5% bovine serum albumin (BSA) in PBS for 1 h and subsequent incubation with 1:500 rabbit polyclonal anti-NF-kκB p65 antibody (06-418, Sigma Aldrich, St. Louis, MO, United States) incubation in 1% BSA in PBS for 1 h at room temperature. Secondary antibody Alexa flur-488 anti-rabbit at 1:2000 (Life Technologies, Waltham, MA, United States) incubation were performed for 1 h at room temperature. The cells were then washed with PBS and counterstained with DAPI at 1:5000 (Calbiochem, Billerica, MA, United States) for 5 min. The slides were mounted with ClearMount with Tris buffer (Electron Microscopy Sciences, Hatfield, PA, United States) before imaging with confocal microscopy (Nikon C1 Plus Ti Eclipse epi-fluorescence). The effects of treatment with cffDNA and LPS were for 30 mins and were measured as percent NF-κB p65 nuclear translocation, and quantified using immunofluorescent imaging.

### Immunohistochemistry

The fetal membrane tissues that were subjected to 30 mins, 1 h, and 2 h of control (no DNA), whole (non-sonicated), and sonicated cffDNA treatment were placed in 10% formalin for 24 h before being stored in sterile PBS. The fetal membranes were embedded in paraffin and cut tissue sections (5 µM) mounted on charged microscope slides. Immunohistochemistry was performed according to the Vectastain ABC immunoperoxidase staining manufacturer’s protocol (*n* = 8) using 1:100 rabbit polyclonal NF-κB p65 primary antibody (06-418, Sigma Aldrich, St. Louis, MO, United States) and the 3,3′-Diaminobenzidine (DAB) peroxidase HRP substrate (Vector Laboratories, Burlingham, CA, United States). The sections were then re-dehydrated and mounted with Permount (Fisher Chemicals, Pittsburg, PA, United States).

### IL-6, TNF-α, and GM-CSF ELISA

All cytokine secretion was assessed from condition media samples collected from the top and bottom well of the Transwell explant system. The Human IL-6, TNF-α, and GM-CSF Quantikine ELISA Kits (R&D Systems, Minneapolis, MN, United States), were all used following the manufacturer’s protocol. The secreted protein levels were normalized to total protein concentrations for each sample, measured by protein assay compatible with conditioned media (Pierce, Waltham, MA, United States).

### Zymography

The condition media collected from the top and bottom wells of the fetal membrane explant system were assessed for pro-matrix metalloproteinase-2/9 (pro-MMP2, pro-MMP9) activity. The condition media (5 µg) samples were mixed with sodium dodecyl sulfate-polyacrylamide gel electrophoresis (SDS PAGE) sample loading buffer. The samples were run on a 10% SDS PAGE gel containing 1 mg/ml gelatin and separated by electrophoresis. The resolving gel was incubated in an assay buffer (40 mM Tris, 0.2 M NaCl, 10 mM CaCl2, 0.1 µM zinc chloride, pH 8.8) overnight at 37°C in a shaking incubator. After incubation, the transfer gels were exposed to a staining buffer (methanol, acetic acid, dH2O, and Coomassie blue) at room temperature while shaking for 45 min. They were then rinsed with dH2O until excess staining solution was removed. The gels were incubated with destaining solution (methanol, acetic acid, and dH2O) until the bands could clearly be seen. The clear bands on the gel indicated the area of enzyme activity. The clear bands on the gel were quantified using ImageJ (Rasband, 2018).

### DNA Sex Determination

Polymerase chain reaction (PCR) based on sex determination was performed to identify the presence of sex region y gene (SRY) for males or alanine aminotransferase-1 gene (ALT1) for females. The sequences of primers for SRY were 5′-CAT​GAA​CGC​ATT​CAT​CGT​GTG​GTC-3′, 5′-CTG​CGG​GAA​GCA​AAC​TGC​AAT​TCT T-3′ and 5′-CCC​TGA​TGA​AGA​ACT​TGT​ATC​TC-3’, and 5′-GAA​ATT​ACA​CAC​ATA​GGT​GGC​ACT-3′ for ATL1 (Settin et al., 2008). Each PCR reaction comprised 100 ng DNA, 1X PCR buffer minus Mg, 0.2 mM dNTP mixture, 1.5 nM MgCl2, 0.5 µM SRY and ALT1 primers (FWD and REV), and 2.5 units Taq DNA Polymerase (5 U/µl) (Invitrogen, Waltham MA, United States). All PCR reactions were performed in a thermal cycler (PTC-225, Peltier Thermal Cycler, MJ Research, NH, United States) at 94°C (3 min) for initial DNA denaturation, followed by 35 cycles of 94°C (15 s) for DNA denaturation, 55°C (30 s) for primer annealing and 72°C (90 s) for primer extension, with a final extension of the cycle at 72°C (10 min). The amplified PCR products were separated on a 2.5% agarose gel with ethidium bromide and imaged under UV transillumination. The product size of SRY was 254 bp for the Y chromosome and ALT1 was 300 bp for the X chromosome. Two product bands at 254 bp and 300 bp identified male samples and one band at 300 bp female samples.

### Methylation Status Quantification

The percent DNA methylation status of cffDNA samples was calculated and quantified using MethylFlash Global DNA Methylation (5-mC) ELISA Easy Kit (colorimetric) according to the manufacturer’s protocol (Epigentek, Farmingdale, NY, United States).

### Statistical Analysis

All statistical analysis was performed using GraphPad Prism 6.0 (GraphPad Software, San Diego, United States). The data throughout the figures are expressed as ± standard error of the mean (SEM). All statistical comparisons between the groups were identified by using one-way ANOVA analysis followed by Bonferroni’s multiple comparison tests or by paired t-tests, appropriately. The correlation statistics were performed using Pearson’s correlation coefficient calculation. The differences *p < 0.05 and **p < 0.01 were considered statistically significant.

## Results

### Sonication Breaks Cell-Free Fetal DNA Into Smaller Fragments but Does Not Decrease the Percentage of Methylation of the DNA

**FIGURE 1:**
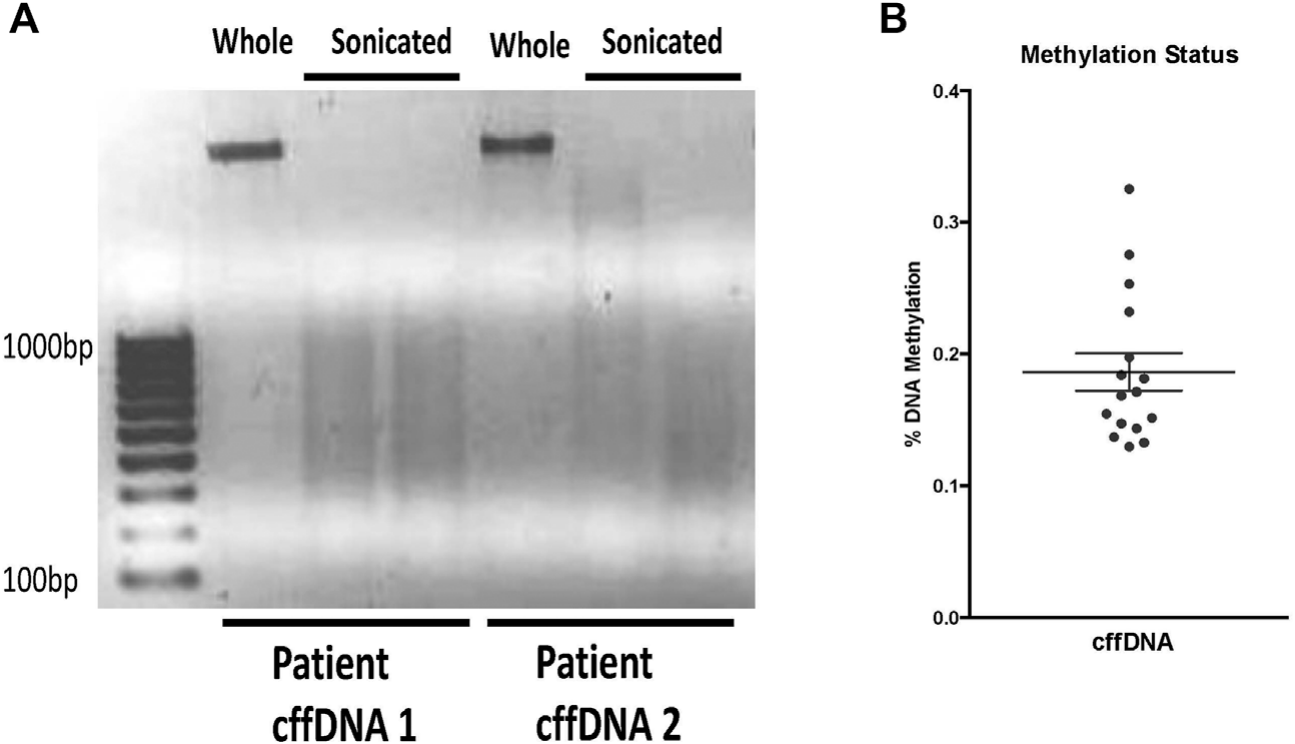
Characterization of fetal DNA fragments isolated from AECs. **(A)** Ethidium bromide stained gel of DNA either; whole—the cffDNA upon immediate isolation from primary human amnion epithelial cells or, sonicated—cffDNA isolated from primary human amnion epithelial cells but sonicated for 4 min. Two different patient samples of fetal DNA are shown as examples. **(B)** Quantification of methylation percentage of DNA from the individual patients’ collected FM samples. Each dot indicates separate patients’ DNA *n* = 16.

In order to study the potential of fetal DNA to stimulate inflammation in the human FM, we first sought to obtain DNA fragments more consistent with the sizes of DNA fragments found in AF. The non-sonicated whole DNA (w.cffDNA) freshly isolated DNA from AEC contained much larger >1000 bp fragments (Figure 1A). While the DNA that was sonicated for 4 min (4 m.s cffDNA) broke into fragments that ranged from 100 to 1000 bp. As it has also been shown *in utero* that DNA fragments released from cells *via* apoptosis or necrosis are often hypomethylated (Gordevičius et al., 2020), we assessed the methylation status of the cffDNA. The methylation ranged from 0.1% to 0.4% (Figure 1B) but this did not decrease after 4 min of sonication as no statistical difference in the percentages of methylation was seen when the DNA was sheared into smaller fragments (variation between the samples before and after sonication ± 5%–10%, which was within the range of assay variation for technical repeat samples).

### Fetal DNA Causes the Nuclear Translocation of the NFκB p65 Subunit in Both Isolated Human Amnion Epithelial Cells and in Cells Throughout the Layers of Fetal Membrane Explants

**FIGURE 2:**
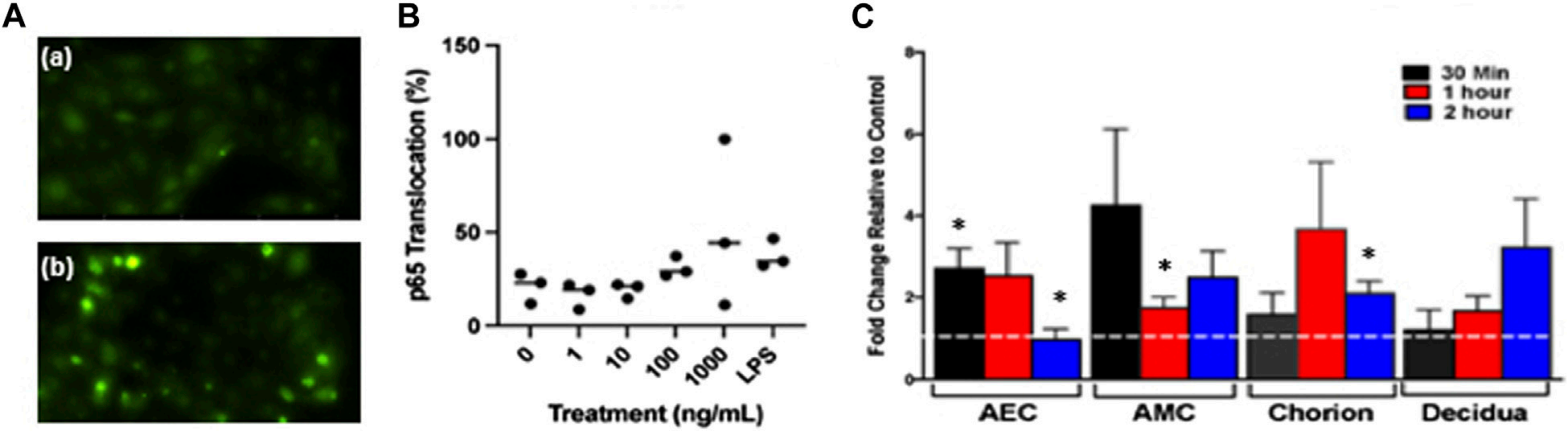
Fetal DNA causes the translocation of the NF-κB subunit p65. **(A)** Immunocytochemistry of the nuclear translocation of p65 **(a)** not treated with cffDNA and **(b)** treated with 100 ng/ml of 4 m.s. cffDNA. Green—p65. **(B)** Quantitation of the percentage of p65 translocation in AECs treated with 1, 10, 100, and 1,000 ng/ml 4 m.s. cffDNA or LPS 1000 ng/ml for 30 min *n* = 3 AEC from different patients’ isolated cells. **(C)** Fetal membrane explants treated only apically with 100 ng/ml 4 m.s. cffDNA for 30 mins, 1, and 2 h. The dotted white line represents untreated control, AEC—amnion epithelial cells. AMC—amnion mesenchymal cells. The data displayed as fold change from no treatment control. (*n* = 3 different patients’ fetal membrane explants). *p = < 0.05.

Following the isolation and characterization of fetal DNA, we treated isolated primary human AECs with 4 m.s cffDNA to determine if it would cause the translocation of the inflammatory transcription factor, NFκB. After immunocytochemistry to visualize the NFκB p65 subunit (Figure 2A), the nuclear location was clearly seen at low levels in untreated cells (22.91%). However, after only 30 min of treatment with 4 m.s cffDNA, quantitation of the NFκB p65 subunit nuclear translocation (Figures 2A,B) after treatment with the higher concentrations of cffDNA (100 and 1,000 ng/ml) was 29.16% and 43.75% respectively (Figure 2B). This response was similar to that seen with 100 ng/ml of LPS (34.37%).

As we detected an increase in the nuclear translocation of NFκB p65 within individual isolated AECs after treatment with 4 m.s cffDNA, we treated human FM explants held in transwell holders with 100 ng/ml of 4 m.s cffDNA just to the apical side of the explant. This was because we wanted to determine specifically if the apical treatment of the FM explant with the 4 m.s cffDNA, would be able to propagate a signal from the amnion surface of the FM to the underlying cells over time. This strategy was conceived as the fetal DNA source we were seeking to study was that which would originate in the AF. An increase in the translocation of nuclear NFκB p65 (compared to no treatment control) observed after only 30 min of apical treatment of the FM explant was statistically significant in the AEC (171% *p* = 0.023) (Figure 2C). This translocation was sustained over 1 h (153.23% *p* = 0.05) compared to no treatment control but after 2 h had returned to untreated explant control levels. Although the 4 m.s cffDNA was only applied to the AEC (the apical side of the FM explant), translocation was also seen in the amnion mesenchymal cells (AMC) after 30 min (324.98%). This was reduced after 1 h (74.88% *p* = 0.041) but remained higher than baseline for the remainder of the 2 h tested (149.06% *p* = 0.07) (Figure 2C). In addition, the cells of the chorion under the AMC also showed translocation of NFκB p65 compared to the untreated explants but this appeared to take 1 h to reach levels higher than that seen at baseline (267.73%). This then began to return toward untreated levels after 2 h but due to low interpatient variation was a statistically significant difference (110.24% *p* = 0.02 compared to untreated control). Finally, p65 nuclear translocation at levels higher than the untreated explants was also seen in the cells of the decidua (under the chorion) but this only became apparent after 2 h and this increase did not reach significance (222.06%) of treatment (Figure 2C). Thus, a significant increase in NF-κB p65 nuclear translocation was seen in all of the layers of the FM except the decidua, (although this may have occurred if the FM were treated for longer times), appearing to take longer for the translocation to peak the further away from the treated surface the cells were in the FM explant model (Figure 2C).

### Effect of Fetal DNA on the Secretion of Proinflammatory Cytokines and Matrix Metalloproteinases From Fetal Membrane Explants

**FIGURE 3:**
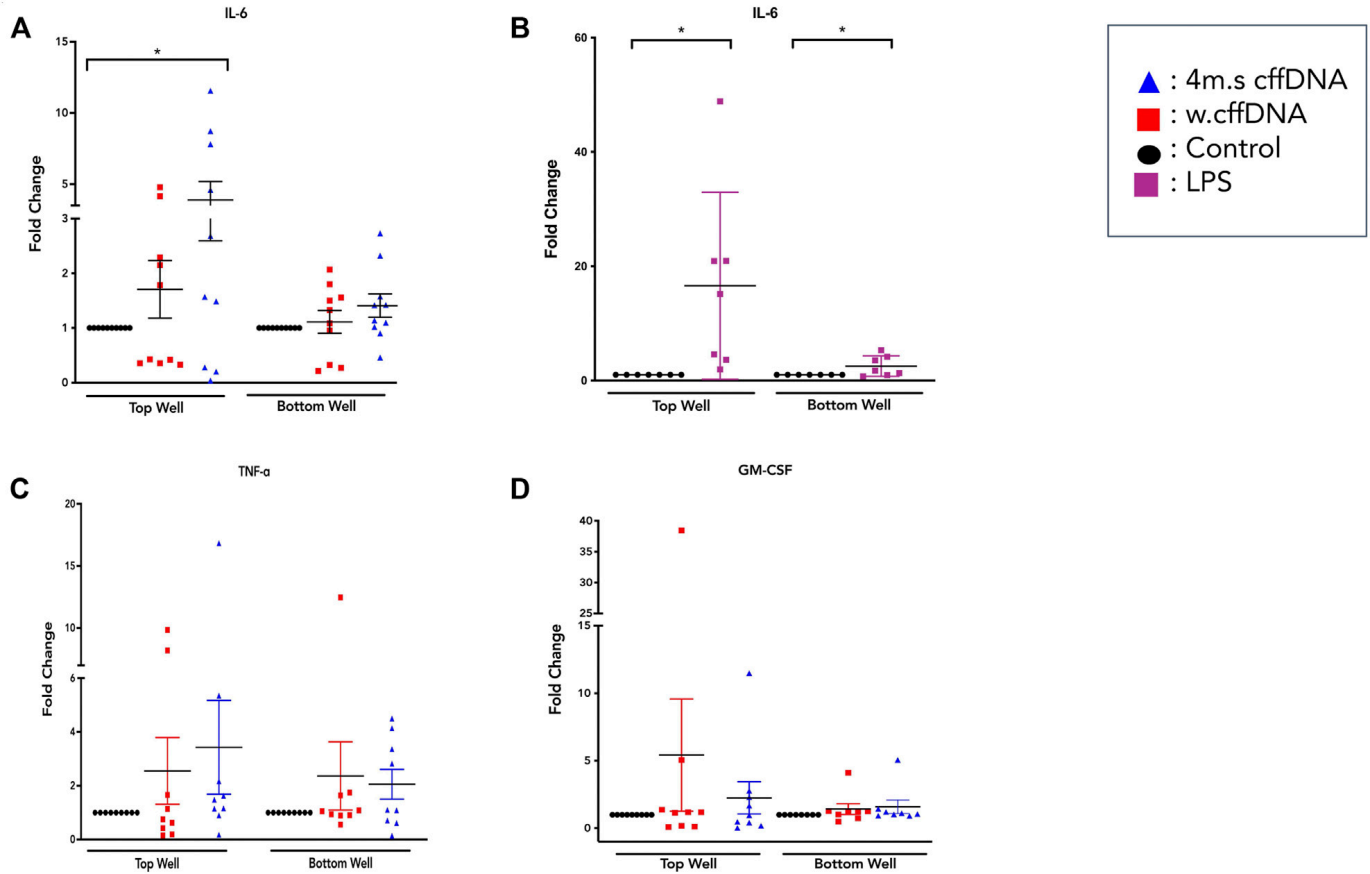
Cytokine secretion from fetal membrane explants after treatment with w.cffDNA or 4 m.s cffDNA. Fold change secretion, compared to no treatment control, of cytokines into the apical (top) well or basal (bottom) well of the transwell fetal membrane explant after treatment with (red) w.cffDNA or (blue) 4 m.s. cffDNA at 100 ng/m. **(A)** IL-6, **(B)** IL-6 after treatment with LPS (100 ng/ml), **(C)** TNF-ɑ, and **(D)** GM-CSF. *n* = 7–10 patients. *p = < 0.05.

As we were able to see an increase in the translocation of NF-κB p65 to more nuclei in cells from the amnion and cells throughout our FM explant model after treatment with cffDNA, we tested if this stimulus was also able to increase the secretion of cytokines that are important for FM weakening at the end of gestation. However, first we measured the levels of activity of lactate dehydrogenase from the media from the apical and basal wells of our explant, over the total 48 h time taken for our experiment. This was performed to test the health of the tissue from collection to the end of treatment with fetal DNA. No changes in the level of lactate dehydrogenase were seen over the 48 h, or due to the addition of fetal DNA (data not shown). Despite the FM explant being treated only on the apical side of the explant with fetal DNA for 24 h, IL-6 secretion was increased into both apical and basal compartments with both 4 m.s.cffDNA (fold change 3.05 apical, 1.39 basal, respectively) and w. cffDNA (fold change 1.70 apical, 1.13 basal, respectively) (Figure 3A). However, the increase in secretion only reached statistical significance in the apical wells treated with 4 m.s cffDNA (*p* = 0.038) compared to the untreated control (Figure 3A). Although the NF-κB p65 translocation response seen in the cells of the amnion was similar for both the cffDNA and LPS, the IL-6 secretion following the treatment with LPS treatment caused a much higher increase in IL-6 secretion than that seen with cffDNA (fold change, apical 16.55, basal 2.52, *p* = 0.02 and *p* = 0.04, respectively) (Figure 3B). It was interesting to note that the LPS only caused a small increase in IL-6 secretion into the basal compared to the apical media compartment. A non-significant increase in TNF-α secretion level was seen after treatment with both 4 m.s (fold change apical 3.43, basal 2.05) and w. cffDNA (fold change apical 2.55, basal 2.36) into the apical and basal media (Figure 3C). Non-significant increases in GM-CSF secretion (fold change apical 2.25, basal 1.59) were also observed (Figure 3D).

**FIGURE 4:**
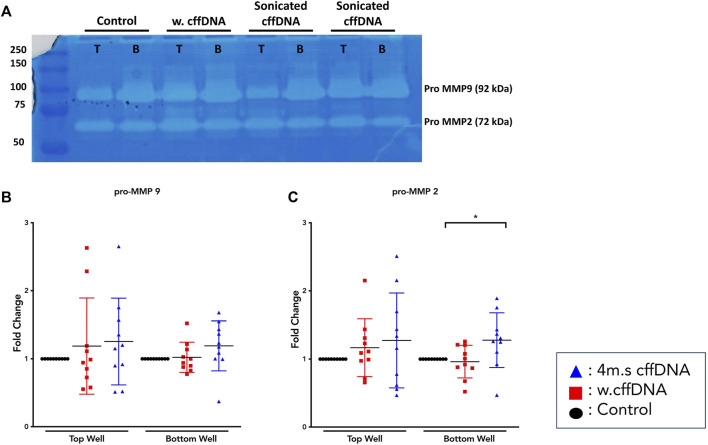
Matrix metalloproteinase activity from fetal membrane explants after treatment with w.cffDNA or 4 m.s. cffDNA. The fold change activity, compared to no treatment control, of MMPs from the apical (top) well or basal (bottom) well of the transwell fetal membrane explant after treatment with (red) w.cffDNA or (blue) 4 m.s cffDNA at 100 ng/ml. **(A)** Example zymography gel. T = apical, B = basal, **(B)** Pro-MMP9, and **(C)** pro-MMP2. *n* = 10 patients. *p = < 0.05.

Because our data showed that the fetal DNA was able to increase the activation of NF-κB and increase the secretion of some of its downstream cytokines, we also wanted to determine whether it was able to increase the levels of the enzymes that are crucial to the degradation of the extracellular matrix. Our MMP activity assay data showed that some patients’ explants did show non-significant increases in activity of pro-MMP9 (Figure 4A,B), especially into the apical well. The results for pro-MMP2 (Figure 4C) showed a more consistent increase in activity after all fetal DNA treatment conditions, reaching significance after apical 4 m.s cffDNA treatment (fold change 1.27 *p* = 0.04) of the enzyme into the basal media compartment.

### The Effect of Fetal Sex on the Fetal Membrane Explant Responses to cffDNA

**FIGURE 5:**
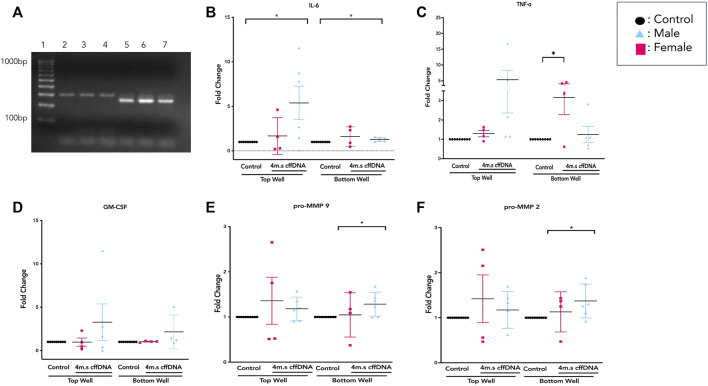
Sex-specific response of FM explants to 4 m.s cffDNA. **(A)** Example of PCR product gel shows that in lanes 2–4 the expression of SRY indicates a female fetus, whereas the expression of both SRY and ALT1 in lanes 5–7 indicated a male fetus. The fold change secretion, compared to no treatment control, of cytokines into the apical (top) well or basal (bottom) well of the transwell fetal membrane explant after treatment with 4 m.s cffDNA at 100 ng/ml. **(B)** IL-6, **(C)** TNF-alpha, and **(D)** GM-CSF **(E)** pro-MMP9 **(F)** pro-MMP2. Black—untreated control. Blue- male fetus, and pink—female fetus. *n* = 7–10 patients. *p = < 0.05 **p = < 0.001.

**FIGURE 6:**
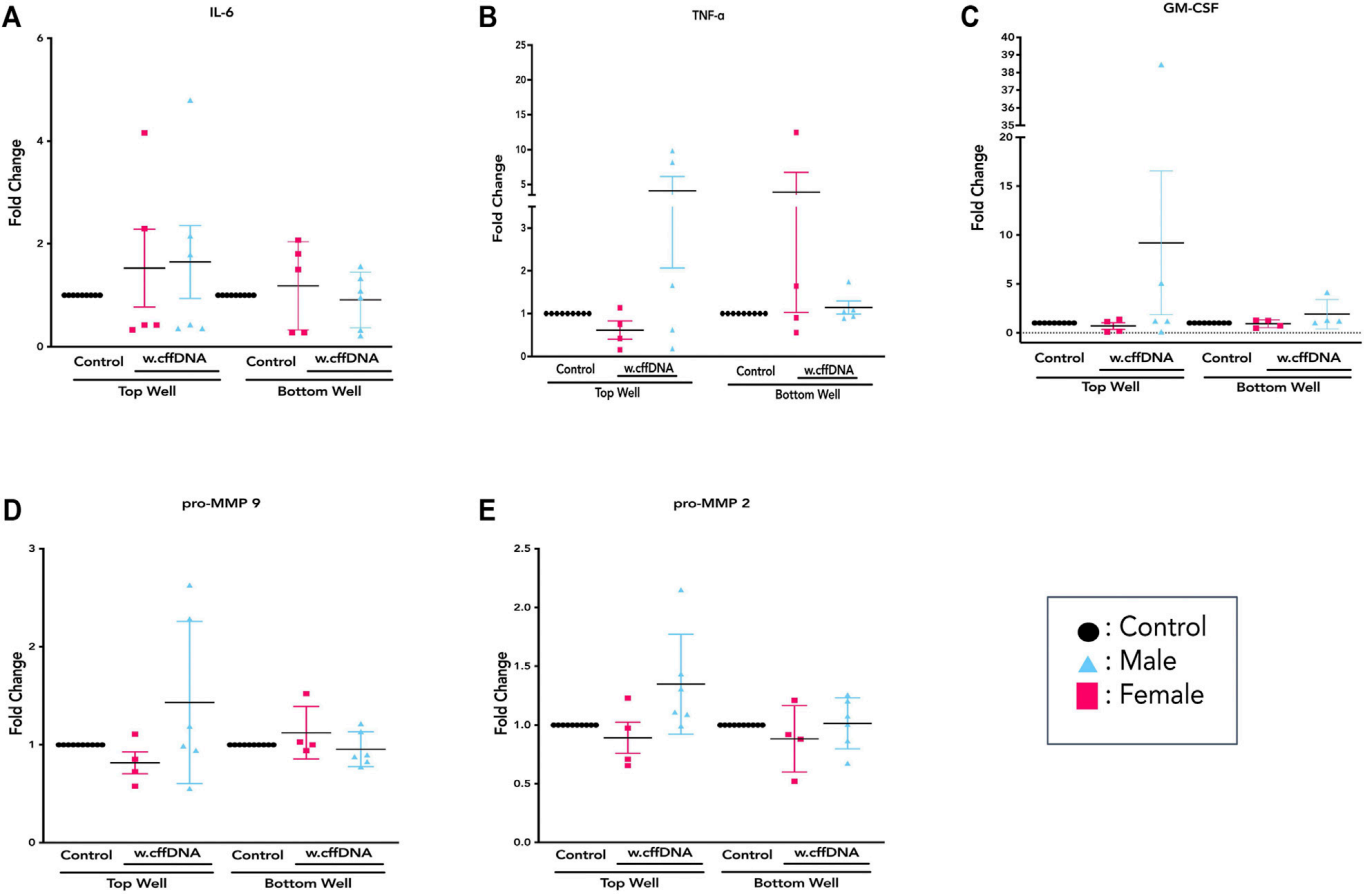
Sex-specific response of FM explants to w.cffDNA. The fold change secretion, compared to no treatment control, of cytokines into the apical (top) well or basal (bottom) well of the transwell fetal membrane explant after treatment with w.cffDNA at 100 ng/ml. **(A)** IL-6, **(B)** TNF-alpha, and **(C)** GM-CSF **(D)** pro-MMP9 **(E)** pro-MMP2. Black—untreated control. Blue- male fetus, and pink—female fetus. *n* = 7–10 patients. *p = < 0.05 **p = < 0.001.

The data from the treated FM explants showed large interpatient variation throughout the measurement of cytokine secretion and MMP activity. Therefore, as it is known that fetal sex can alter the inflammatory response (Mitchell et al., 2017; Velten et al., 2018; Allard et al., 2019) we determined the sex of each fetus for the tissues we used for this series of experiments. The males were identified by the dual expression of SRY and ALT1 expression, whereas the females only expressed the X-linked SRY gene (Figure 5A). As the response to the cffDNA that had been sonicated for 4 min appeared to be the more robust for several of our targets of interest, we first analyzed this data by separating the male and female responses (Figures 5B–F) and then repeated this analysis strategy for the w. cffDNA (Figures 6A–E), to uncover any sex-specific patterns of response.

Overall, it was clear that the FM explants from pregnancies with a male fetus had a larger cytokine response than those with females to 4 m.s cffDNA; secreting more IL-6 into both the apical (fold change 5.38, *p* = 0.041) and basal (fold change 1.28, *p* = 0.011) wells (Figure 5B). Similar to IL-6, the males also appeared to have more of an increase in GM-CSF secretion into both apical (fold change 3.27) and basal (fold change 2.16) wells but this did not reach significance (Figure 5D). When the data was reviewed based on sex for the pro-MMP activity, both activities of pro-MMP9 and pro-MMP2 were statistically significantly higher in males (fold change pro-MMP9 1.28, pro-MMP2 1.37; *p* = 0.02 and *p* = 0.03) from the basal wells (Figures 5E,F). Interestingly, this was not the case for TNF-α, as although some of the males clearly increased their secretion of this cytokine, especially apically, only the FM explants from female fetuses significantly (fold change 3.15; *p* = 0.04) increased their TNF-α secretion into the basal well (Figure 5C). When the data was then analyzed from the FM explants treated with w. cffDNA, no significant differences were seen for males or females, for any of the cytokines secreted, or for the activity levels of the pro-MMPs (Figure 6). However, several of the targets were (non-significantly) increased from the male FM explants, particularly those measured from the apical wells. Thus, collectively this analysis of the data separated by fetal sex (Figures 5, 6) illustrated that the male and female FM tissues responded differently.

### The Effect of the Level of Methylation on the Fetal Membrane Explant Response to cffDNA

The initial characterization of the sonicated and non-sonicated fetal DNA (Figure 1) showed that there was some variation in the percentages of methylation from different patients. Thus, we also sought to evaluate the influence of the percentage of methylation on our treatment response to fetal DNA. We wanted to determine if this would affect the FM response, but also to see if any difference would, in part, explain the variation between the male and female responses.

**FIGURE 7:**
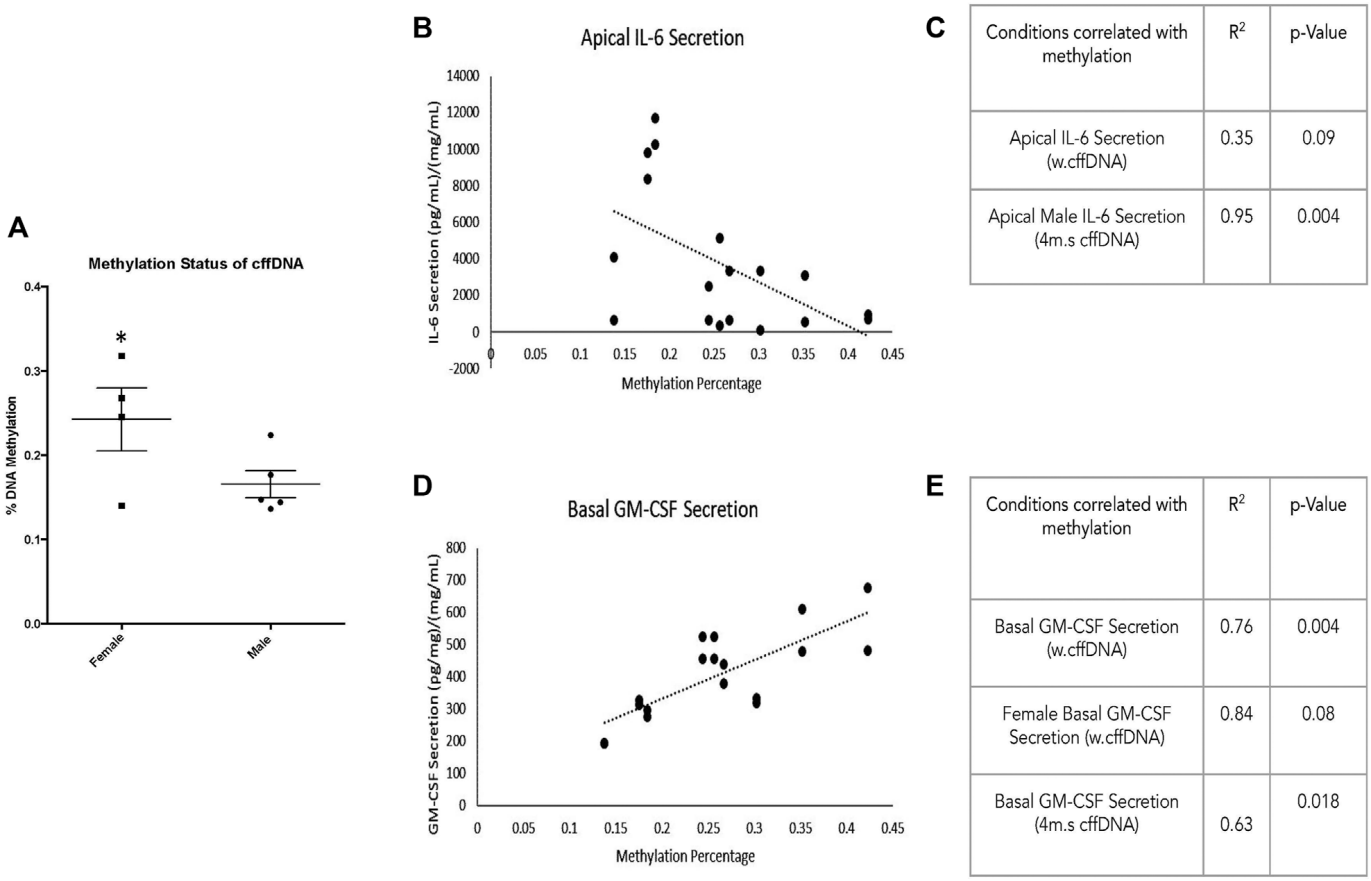
Fetal membrane response to fetal DNA relative to methylation percentage. **(A)** Percentage methylation of male and female fetal DNA. The correlation of cytokine secretion from FM correlated with percentage methylation of fetal DNA used for apical treatment. **(B)** Apical secretion of IL-6 regardless of fetal DNA fragment size. **(C)** Table illustrating the correlation between the percentage of methylation and the apical secretion of IL-6 with w.cffDNA; the apical secretion of IL-6 from the male explants with 4 m.s cffDNA. **(D)** Basal GM-CSF secretion regardless of fetal DNA size is used for treatment. **(E)** Table illustrating the correlation between the percentage of methylation and the basal secretion of GM-CSF of FM treated with w. cffDNA; the basal GM-CSF secretion of FM treated with 4 m.s. cffDNA; the basal secretion of GM-CSF from the female explants treated with w.cffDNA.

When the percentage of methylation for all of the male fetal DNA was compared to the female fetal DNA, they had a significantly lower (*p* = 0.040) percentage of methylation (0.16%, 0.24% respectively) (Figure 7A). Next, we took our cytokine secretion and our pro-MMP activity data and for each sample correlated the response with the percentage of methylation. We looked at a number of correlations with the percentage methylation; the specific target of interest in the apical or the basal well irrespective of fetal DNA type, the target in the apical or basal also split by fetal DNA type (w.cffDNA or 4 m.s. cffDNA), the target in the apical or basal divided into male and female and also the cffDNA type. A complete record of all correlations performed, regardless of statistical significance, is documented in Supplementary Data Table S1. When we correlated the IL-6 secretion into the apical well, regardless of cffDNA type or fetal sex (Figure 7B) we found a significant negative correlation with the percentage of methylation (*p* = 0.019). In addition, we found that there was a strong negative correlation for IL-6 secreted into the apical well (Figure 7B) with w. cffDNA (*p* = 0.09) and that when this was analyzed for just the male fetus FM with 4 m.s cffDNA (Figure 7C) it was statistically significant (*p* = 0.004). Thus, when the percentage of methylation is lower, more IL-6 was secreted into the apical well, particularly from the male FM when treated with 4 m.s cffDNA (Figures 7B,C).

Interestingly, the other cytokine that correlated with the percentage of methylation was GM-CSF (Figure 7D). When the data were combined for both types of cffDNA and both fetal sexes (Figure 7E), a positive correlation was seen for GM-CSF in the basal well (*p* = 0.00012). In addition, when the cffDNA type was separated, both the 4 m.s (Figure 7E) and w. cffDNA (Figure 7E) treatments caused GM-CSF secretion into the basal well that positively correlated with the percentage of methylation (*p* = 0.018 and *p* = 0.004 respectively). Finally, when the data was separated into the two sexes and the cffDNA type, a strong correlation was seen for the female FM explant (Figure 7E) secretion of GM-CSF into the basal well with w.cffDNA (*p* = 0.08). Thus, when the percentage of methylation is higher, more GM-CSF is secreted into the basal well, which may be stronger than the female FM treated with w.cffDNA (Figure 7E).

## Discussion

It is known that cffDNA can stimulate inflammation in several tissues and cell types (Goldfarb et al., 2018; Kazemi et al., 2021; Yeganeh Kazemi et al., 2021) but its effects have not yet been studied on the human FM. In addition to this, it is also known that the sex of the fetus can influence both the magnitude and the resultant cytokine profile of the inflammatory response, depending on the specific circumstances (Burns et al., 2015; Mitchell et al., 2017). Therefore, as the AF’s concentration of fetal DNA increases with gestational age (Park et al., 2021), we sought to understand its effect(s) on the human FM. Together our data support the role of fetal DNA as an inflammatory stimulus in the FM, as measured by increased NF-κB translocation and cytokine secretion. However, our data also suggest that this response is different in FM tissues from male and female fetus pregnancies and that both the fragment size and methylation status of the fetal DNA could influence the magnitude and type of molecule secreted.

**FIGURE 8:**
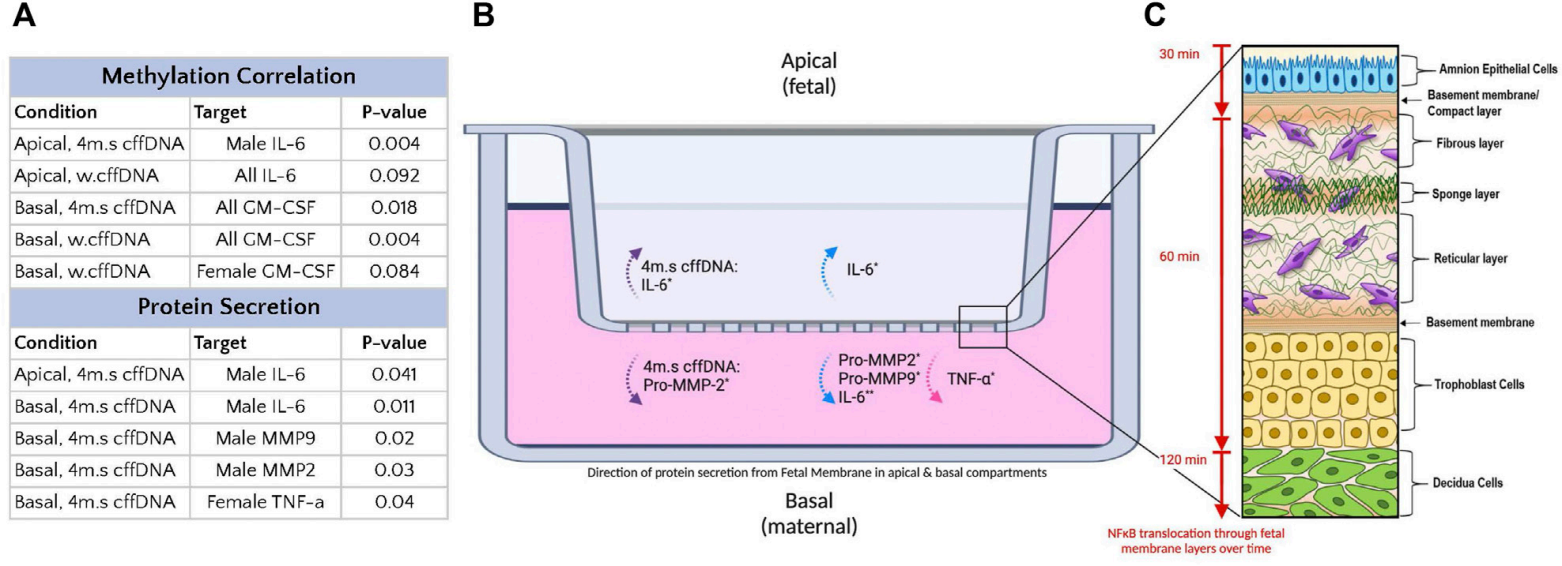
Summary of the effect of fetal DNA on fetal membrane explants from male and female fetus pregnancies. **(A)** Table of treatment conditions, secretion locations with DNA species causing a response. **(B)** Transwell system with fetal membrane explant. The secretion into the apical media compartment of IL-6, and pro-MMP-2, into the basal media compartment of IL-6, TNF-α, pro-MMP2, and pro-MMP9. The dotted purple arrow indicates secretion regardless of fetal sex. The dotted blue arrows indicated secretion by male fetus FM. The dotted pink arrows indicated secretion by female fetus FM. *n* = 7–10 patients. *p = < 0.05 **p = < 0.001. **(C)** Diagram of fetal membranes (modified from Richardson et al., 2020) with attached decidua, illustrating the wave of NF-κB p65 subunit translocation to the nucleus over time (amnion→chorion→decidua) in response to fetal DNA treatment (apical).

The proinflammatory transcription factor NF-κB has been shown to be important to drive inflammatory cascades at the end of pregnancies in the myometrium, cervix, and FM (Gomez-Chavez et al., 2021). Thus, it is considered central to what understand about the progression of normal parturition. Interestingly, the NF-κB dimer can be activated by many different receptors signaling cascades that result in the phosphorylation of its inhibitory protein IκB (Schiedereit, 2006), this activated dimer then causes the transcription of a whole cadre of cytokines (Lui et al., 2017) that are key for parturition including; IL-1β, IL-6, IL-8, TNFα, and IFN-γ (Burns et al., 2015). The consequence of this is that NF-κB is able to influence a wide range of cellular pathways, in addition to inflammation, including cell survival, proliferation, and angiogenesis (Lui et al., 2017). Our data clearly shows the propagation of the translocation of its p65 subunit to the nucleus in cells through the FM with time (Figures 2, 8). This illustrates the potential capacity of stimulating factors in the AF to activate this transcription factor, leading to wide-ranging downstream consequences. Interestingly, this means that factors in the AF could also readily lead to changes below the AEC, affecting the ability of AMC to regulate the ECM in the amnion and hence its strength. They could also influence the behavior of the chorion and decidua in a variety of ways. Our data in the layers of the FM also demonstrated the known trafficking behavior of this transcription factor; as it quickly translocates to the nucleus when its inhibitory protein (IκB) is phosphorylated, and then out again, as this inhibitor is *de novo* synthesized. This results in pulsatile waves of activation and deactivation of this transcription factor (Klinke et al., 2008). This propagation of signaling through the tissue is also supported by the detection of increased levels of the cytokines, IL-6 and TNFα and both pro-MMP 2 and pro-MMP 9, in the basal compartment explant media (Figure 8), despite this side of the membrane not being treated.

Although *ex vivo* FM explants lack the immune cells that are crucial for a full understanding of how this tissue behaves at the end of pregnancy, they still enable the study of the resident cell types and their complex interactions. As there is currently no reliable test to determine the proximity to labor, experiments with these tissues often exhibit large interpatient variation. This can also come from inaccuracies in the estimation of gestational age and the normal variation in the immune responses between individuals (Klein and Flanagan, 2016). This leads to experiments with FM tissues that may seem to have little response to treatment due to factors like increasing levels of cellular senescence or apoptosis with age (Reti et al., 2007; Menon et al., 2019). Thus, without a legitimate way to dismiss these results, they should be included in data analysis. Newly developed models like the fetal membrane organ on a chip (Richardson et al., 2020), coupled with *ex vivo* FM explants, and may help us to further understand some nuances of cell-to-cell interactions in this tissue.

Despite the variation in our FM responses, we saw many samples were clearly very responsive, and the disaggregation of the data based on fetal sex was illuminating. We saw that male and female FM both led to the increased secretion of cytokines and pro-MMPs but that this response varied between the sexes. The others have shown that males have a more robust inflammatory response but other studies have also shown that females, in other circumstances, have a more robust response than males (Burns et al., 2015; Mitchell et al., 2017). This might be explained by the variety of the targets measured between the studies (Makinson et al., 2017; Wegner et al., 2017; Allard et al., 2019; Na et al., 2021; Goldstein et al., 2021). We saw that the species of fetal DNA also caused differential responses between the sexes (Figure 8). Overall, the male FM seemed to have a larger response to the male fetal DNA, especially when it was sonicated into smaller fragments. We also saw that the male fetal DNA was generally more hypomethylated (Figure 7A). This supports other reports that have demonstrated that in different cell and tissue types that the X chromosome in females was hypermethylated compared to the males (Bianca-Miotto et al., 2016; Garcia-Calzón et al., 2018). However, the cause for this is not understood. Although the females in our study generally secreted lower levels of cytokines than the males, they did have a significant increase in basal TNF-α secretion, a small increase in the pro-MMPs, and an increase in GM-CSF that correlated well with the higher levels of methylation of female fetal DNA (Figure 8). Thus, both males and females did increase their section of proinflammatory cytokines and pro-MMPs, although their responses were different. An increased IL-6 response by the males might precipitate a strong inflammatory response driven by JAK/STAT and MAPK signaling (Rose-John, 2021), whereas the increased TNF-α response in females may more likely lead to further NF-κB activation. Thus, although both cytokines are themselves increased through NF-κB pathway activation, they may result in different pathway activation and consequences. Indeed, it has been shown in other tissues that TLR activation rapidly results in TNF release which is then followed by IL-6, and that blocking TNF decreases IL-6 levels (Ghezzi et al., 2000). Therefore, future work should focus on understanding the differential responses and how these may change and influence specific pathological processes during pregnancy.

Our results are composite responses influenced by both sex-specific FM tissues, coupled with sex-specific fetal DNA from the same pregnancy. This is different from the studies that aimed to understand the role of cffDNA in maternal circulation because these studies always measure the female response to either male or female cffDNA, while our tissue and DNA are sex-matched. However, a limitation of our study is that although we are using FM and fetal DNA from the same pregnancy, we did not isolate cffDNA directly from the AF. Thus, there may be differences between our DNA and AF DNA that are not based on fragment size or methylation status, and that have not been fully characterized to date. cffDNA is thought to be liberated *via* the processes of apoptosis and necrosis, and only recently have studies begun to sequence circulating fetal versus maternal cffDNA to further understand its specific characteristics (Enninga 2022).

Originally it was thought that our not sonicated “whole” DNA would be unlikely to elicit an FM response and may serve as a good additional negative control in our experiments. However, we did see a response, but this differed from that seen with the sonicated, smaller fragments of fetal DNA. It is thought that *in vivo* small hypomethylated fragments of DNA interact with intracellular TLR9 (Scharfe-Nugent et al., 2012) after it enters the cell *via* a variety of mechanisms (van Boeckel et al., 2018). As we saw an FM response with this w.cffDNA, it may be that this larger DNA could be interacting with receptors such as RAGE or STING that are known to be able to bind larger DNA fragments (van Boeckel et al., 2018). Indeed, it is known that the STING receptor can also work with HMGB1 to augment its function (Lee et al., 2021). Our results demonstrating a positive correlation between the percentage of methylation and GM-CSF and the negative correlation between methylation and IL-6 are intriguing. However, they also support the premise that different forms of DNA, whether based on size, or in this instance methylation status, are able to elicit different responses. This further emphasizes the importance of the study of the interaction of these different species with potential receptors to improve our understanding of fetal DNA as a signaling molecule in pregnancy.

Collectively, our data also highlight the need to improve our understanding of the influence of having a male or female fetus in normal pregnancy and also those with negative outcomes. They also suggest that the fetal DNA that builds up in the AF with gestational age may signal to the cells of the FM and potentially the underlying maternal tissues. Indeed, it may be able to contribute to the inflammatory load that builds to initiate parturition. Currently, it is not understood how the different fetal DNA fragment sizes or methylation level contributes to this, but it may be through differential receptor interaction and activation. It is also clear that although this DNA is able to activate inflammation in the FM it may only contribute to it once the functional progesterone block is removed from NF-κB (Gomez-Chavez et al., 2021). Thus, in conclusion, our data suggested that fetal DNA can be proinflammatory but the magnitude of response and the resultant downstream signaling molecules is dependent on the sex of the fetus and the specific characteristics of the fetal DNA.

## Data Availability

The original contributions presented in the study are included in the article/Supplementary Material; further inquiries can be directed to the corresponding author.

## Glossary

DAB: 3,3′-Diaminobenzidine
DAPI: 4′,6-Diamidino-2-phenylindole
ALT-1: Alanine aminotransferase-1
BSA: Albumin standard
AEC: Amniotic epithelial cells
AF: Amniotic fluid
AMC: Amniotic mesenchymal cells
ANOVA: Analysis of variance
BP: Base pair
BCA: Bicinchoninic acid
CaCl2: Calcium chloride
cffDNA: Cell free fetal DNA
DAMPs: Danger-associated molecular patterns
DMEM/F12: Dulbecco’s Modified Eagle Medium/Nutrient Mixture F-12
ELISA: Enzyme-linked immunoassay
ECM: Extracellular matrix
FM: Fetal membranes
FBS: Fetal bovine serum
4 m.s. cffDNA: Four minute sonicated cell-free fetal DNA
GM-CSF: Granulocyte macrophage colony-stimulating factor
HSP: Heat shock proteins
HMGB1: High mobility group box protein 1
HRP: Horseradish peroxidase
IHC: Immunohistochemistry
IFNα: Interferon-alpha
IL-1β: Interleukin-1 beta
il-6: Interleukin-6
IAI: Intrauterine infection
JAK: Janus Kinase
LPS: Lipopolysaccharide
MAPK: Mitogen-activated protein kinase
MMP: Matrix metalloproteinase
NF-κB: Nuclear factor-kappa beta
PFA: Paraformaldehyde
PBS: Phosphate buffered saline
PCR: Polymerase chain reaction
RAGE: Receptor for advanced glycation end products
S100A12: S100 calcium-binding protein alpha-12
S100A: S100 calcium-binding protein alpha
SRY: Sex region Y
NaCl: Sodium chloride
SDS: Sodium dodecyl sulfate
SEM: Standard error of the mean
STAT: Signal transducer and activator of transcription
STING: Stimulator of interferon genes
TLR9: Toll-like receptor 9
TNFα: Tumor necrosis factor alpha
w.cffDNA: Whole cell-free DNA.
