# Supplementary material for: Fetal DNA Causes Sex-Specific Inflammation From Human Fetal Membranes
Source: Front Physiol. 2022 Jun 22;13:901726. doi: 10.3389/fphys.2022.901726 (PMC9257279; doi:10.3389/fphys.2022.901726)
Supplement: Supplementary file 1 [file Table1.docx]

Supplementary Table 1: Complete record of correlations of the cytokine secretion with methylation percentage.

| **Molecule** | **Correlative Relationship Tested** | **Coefficient (r)** | **R^2** | **p-value** | **Sample Size (n)** |
| --- | --- | --- | --- | --- | --- |
| TNF-Alpha | 4m.s Top vs Methylation | -0.37379696 | 0.1397 | 0.321691986 | 9 |
| TNF-Alpha | 4m.s Top Female vs Methylation | -0.169776471 | 0.0288 | 0.830223529 | 4 |
| TNF-Alpha | 4m.s Top Male vs Methylation | -0.585746348 | 0.3431 | 0.299371447 | 5 |
| TNF-Alpha | 4m.s Bottom vs Methylation | -0.164654266 | 0.1762 | 0.672055159 | 9 |
| TNF-Alpha | 4m.s Bottom Female vs Methylation | 0.12539314 | 0.0157 | 0.87460686 | 4 |
| TNF-Alpha | 4m.s Bottom Male vs Methylation | -0.425760866 | 0.1813 | 0.474759245 | 5 |
| TNF-Alpha | w.cffDNA Top vs Methylation | -0.419790865 | 0.1762 | 0.260650414 | 9 |
| TNF-Alpha | w.cffDNA Top Female vs Methylation | -0.585502438 | 0.3428 | 0.414497562 | 4 |
| TNF-Alpha | w.cffDNA Top Male vs Methylation | -0.378982557 | 0.1436 | 0.529277932 | 5 |
| TNF-Alpha | w.cffDNA Bottom vs Methylation | -0.340081228 | 0.1762 | 0.370537831 | 9 |
| TNF-Alpha | w.cffDNA Bottom Female vs Methylation | -0.459501601 | 0.1762 | 0.540498399 | 4 |
| TNF-Alpha | w.cffDNA Bottom Male vs Methylation | -0.408668486 | 0.167 | 0.494537002 | 5 |
| pro-MMP9 | 4m.s Top vs Methylation | -0.068201037 | 0.0047 | 0.851502236 | 10 |
| pro-MMP9 | 4m.s Top Female vs Methylation | -0.248735096 | 0.0619 | 0.751264904 | 4 |
| pro-MMP9 | 4m.s Top Male vs Methylation | 0.012578931 | 0.0002 | 0.981132599 | 6 |
| pro-MMP9 | 4m.s Bottom vs Methylation | -0.243536484 | 0.0593 | 0.497752009 | 10 |
| pro-MMP9 | 4m.s Bottom Female vs Methylation | -0.137175449 | 0.0188 | 0.862824551 | 4 |
| pro-MMP9 | 4m.s Bottom Male vs Methylation | -0.32324896 | 0.1045 | 0.532014685 | 6 |
| pro-MMP9 | w.cffDNA Top vs Methylation | -0.23592046 | 0.0557 | 0.511701462 | 10 |
| pro-MMP9 | w.cffDNA Top Female vs Methylation | -0.621328492 | 0.386 | 0.378671508 | 4 |
| pro-MMP9 | w.cffDNA Top Male vs Methylation | -0.107069977 | 0.0115 | 0.840008759 | 6 |
| pro-MMP9 | w.cffDNA Bottom vs Methylation | -0.410422167 | 0.1684 | 0.238760826 | 10 |
| pro-MMP9 | w.cffDNA Bottom Female vs Methylation | -0.595751787 | 0.3549 | 0.404248213 | 4 |
| pro-MMP9 | w.cffDNA Bottom Male vs Methylation | -0.382302469 | 0.1462 | 0.454484039 | 6 |
| pro-MMP2 | 4m.s Top vs Methylation | -0.060527278 | 0.0037 | 0.868080581 | 10 |
| pro-MMP2 | 4m.s Top Female vs Methylation | -0.233712893 | 0.0546 | 0.766287107 | 4 |
| pro-MMP2 | 4m.s Top Male vs Methylation | 0.026827144 | 0.0007 | 0.959768938 | 6 |
| pro-MMP2 | 4m.s Bottom vs Methylation | -0.231884553 | 0.0538 | 0.519158776 | 10 |
| pro-MMP2 | 4m.s Bottom Female vs Methylation | -0.155957597 | 0.0243 | 0.844042403 | 4 |
| pro-MMP2 | 4m.s Bottom Male vs Methylation | -0.302393161 | 0.0914 | 0.560235919 | 6 |
| pro-MMP2 | w.cffDNA Top vs Methylation | -0.167944541 | 0.0282 | 0.642809186 | 10 |
| pro-MMP2 | w.cffDNA Top Female vs Methylation | -0.562580943 | 0.3165 | 0.437419057 | 4 |
| pro-MMP2 | w.cffDNA Top Male vs Methylation | -0.047650331 | 0.0023 | 0.9285786 | 6 |
| pro-MMP2 | w.cffDNA Bottom vs Methylation | -0.219169612 | 0.048 | 0.542940062 | 10 |
| pro-MMP2 | w.cffDNA Bottom Female vs Methylation | -0.257196825 | 0.0662 | 0.742803175 | 4 |
| pro-MMP2 | w.cffDNA Bottom Male vs Methylation | -0.183334381 | 0.0336 | 0.728079499 | 6 |
| GM-CSF | 4m.s Top vs Methylation | -0.4646461 | 0.215896007 | 0.2076 | 9 |
| GM-CSF | 4m.s Top Female vs Methylation | -0.6265725 | 0.392593136 | 0.3734 | 4 |
| GM-CSF | 4m.s Top Male vs Methylation | -0.5871533 | 0.344748957 | 0.2979 | 5 |
| GM-CSF | 4m.s Bottom vs Methylation | 0.79499215 | 0.63201252 | 0.01836 | 8 |
| GM-CSF | 4m.s Bottom Female vs Methylation | 0.80987148 | 0.65589182 | 0.1901 | 4 |
| GM-CSF | 4m.s Bottom Male vs Methylation | 0.77866559 | 0.60632011 | 0.2213 | 4 |
| GM-CSF | w.cffDNA Top vs Methylation | 0.03921778 | 0.001538034 | 0.9202 | 9 |
| GM-CSF | w.cffDNA Top Female vs Methylation | -0.1560259 | 0.024344081 | 0.844 | 4 |
| GM-CSF | w.cffDNA Top Male vs Methylation | 0.30926722 | 0.095646214 | 0.6126 | 5 |
| GM-CSF | w.cffDNA Bottom vs Methylation | 0.87358104 | 0.76314382 | 0.004584 | 8 |
| GM-CSF | w.cffDNA Bottom Female vs Methylation | 0.91566041 | 0.83843399 | 0.08434 | 4 |
| GM-CSF | w.cffDNA Bottom Male vs Methylation | 0.80197759 | 0.64316806 | 0.198 | 4 |
| IL-6 | 4m.s Top vs Methylation | -0.4895821 | 0.239690654 | 0.181 | 9 |
| IL-6 | 4m.s Top Female vs Methylation | 0.00353687 | 0.0000125094 | 0.9965 | 4 |
| IL-6 | 4m.s Top Male vs Methylation | -0.9765475 | 0.953645104 | 0.004296 | 5 |
| IL-6 | 4m.s Bottom vs Methylation | -0.1216593144 | 0.01480098877 | 0.7552 | 9 |
| IL-6 | 4m.s Bottom Female vs Methylation | 0.73684132 | 0.54293513 | 0.2632 | 4 |
| IL-6 | 4m.s Bottom Male vs Methylation | -0.730301802 | 0.533340722 | 0.1612 | 5 |
| IL-6 | w.cffDNA Top vs Methylation | -0.5930488 | 0.351706913 | 0.09234 | 9 |
| IL-6 | w.cffDNA Top Female vs Methylation | -0.7943351 | 0.630968244 | 0.2057 | 4 |
| IL-6 | w.cffDNA Top Male vs Methylation | -0.7791956 | 0.607145724 | 0.1203 | 5 |
| IL-6 | w.cffDNA Bottom vs Methylation | -0.323755274 | 0.1048174774 | 0.3954 | 9 |
| IL-6 | w.cffDNA Bottom Female vs Methylation | 0.65617365 | 0.43056386 | 0.3438 | 4 |
| IL-6 | w.cffDNA Bottom Male vs Methylation | -0.6756604 | 0.45651704 | 0.2106 | 5 |
